# Cord blood myostatin concentrations by gestational diabetes mellitus and fetal sex

**DOI:** 10.3389/fendo.2023.1018779

**Published:** 2023-02-15

**Authors:** Rong Huang, Mark Kibschull, Laurent Briollais, Zdenka Pausova, Kellie Murphy, John Kingdom, Stephen Lye, Zhong-Cheng Luo

**Affiliations:** ^1^ Lunenfeld-Tanenbaum Research Institute, Prosserman Centre for Population Health Research, Mount Sinai Hospital, and Institute of Health Policy, Management and Evaluation, University of Toronto, Toronto, ON, Canada; ^2^ Department of Obstetrics and Gynecology, Mount Sinai Hospital, Temerty Faculty of Medicine, University of Toronto, Toronto, ON, Canada; ^3^ Lunenfeld-Tanenbaum Research Institute, Mount Sinai Hospital, University of Toronto, Toronto, ON, Canada; ^4^ Institute of Health Policy, Management and Evaluation, Dalla Lana School of Public Health, University of Toronto, Toronto, ON, Canada; ^5^ The Hospital for Sick Children, Toronto, ON, Canada; ^6^ Departments of Physiology and Nutritional Sciences, University of Toronto, Toronto, ON, Canada

**Keywords:** gestational diabetes mellitus, myostatin, testosterone, insulin-like growth factor, sex difference

## Abstract

**Introduction:**

Myostatin is a member of the transforming growth factor β superfamily, and is mainly secreted from skeletal muscle. Animal studies have demonstrated that deficiency in myostatin promotes muscle growth and protects against insulin resistance. In humans, gestational diabetes mellitus (GDM) affects fetal insulin sensitivity. Females are more insulin resistant and weigh less than males at birth. We sought to assess whether cord blood myostatin concentrations vary by GDM and fetal sex, and the associations with fetal growth factors.

**Methods:**

In a study of 44 GDM and 66 euglycemic mother-newborn dyads, myostatin, insulin, proinsulin, insulin-like growth factor (IGF)-1, IGF-2 and testosterone were measured in cord blood samples.

**Results:**

Cord blood myostatin concentrations were similar in GDM *vs*. euglycemic pregnancies (mean ± SD: 5.5 ± 1.4 *vs*. 5.8 ± 1.4 ng/mL, P=0.28), and were higher in males *vs*. females (6.1 ± 1.6 *vs*. 5.3 ± 1.0 ng/mL, P=0.006). Adjusting for gestational age, myostatin was negatively correlated with IGF-2 (r=-0.23, P=0.02), but not correlated with IGF-1 (P=0.60) or birth weight (P=0.23). Myostatin was strongly correlated with testosterone in males (r=0.56, P<0.001), but not in females (r=-0.08, P=0.58) (test for difference in r, P<0.001). Testosterone concentrations were higher in males *vs*. females (9.5 ± 6.4 *vs*. 7.1 ± 4.0 nmol/L, P=0.017), and could explain 30.0% (P=0.039) of sex differences in myostatin concentrations.

**Discussion:**

The study is the first to demonstrate that GDM does not impact cord blood myostatin concentration, but fetal sex does. The higher myostatin concentrations in males appear to be partly mediated by higher testosterone concentrations. These findings shed novel insight on developmental sex differences in insulin sensitivity regulation relevant molecules.

## Introduction

Myostatin or growth differentiation factor 8 is a member of the transforming growth factor β superfamily, and is mainly secreted from skeletal muscle (1). Myostatin is a strong negative regulator of skeletal muscle growth (1, 2), while inhibition of myostatin or its signaling prevents fat accumulation and improves insulin sensitivity in mice (3–8). In humans, elevated myostatin levels in skeletal muscle or circulation have been associated with obesity and insulin resistance in adults (9–12). So far, there have been no studies on whether gestational diabetes mellitus (GDM) - a common pregnancy complication (13) that has been associated with impaired fetal insulin sensitivity but enhanced fetal growth (14), may affect cord blood myostatin concentration. Little is known about whether myostatin is associated with fetal growth or fetal growth factors.

Females weigh less than males at birth, despite higher concentrations of major fetal growth factors (insulin and IGF-1), suggesting that females are intrinsically more insulin resistant than males *in utero* (15–17). Given sex differences in fetal growth and insulin sensitivity, we hypothesized that myostatin may exhibit sex differences and correlate with fetal growth. We are aware of only one small study (n=83) on cord blood myostatin which reported no association with fetal sex, and a negative correlation with birth weight (18).

Testosterone promotes protein synthesis, skeletal and muscle growth (19, 20). Higher cord blood testosterone concentrations have been reported in males *vs*. females (21). Decreased testosterone concentrations have been associated with insulin resistance and type 2 diabetes in men (22). Testosterone treatment has been associated with increased myostatin concentrations in men (23). We thus hypothesized that testosterone may mediate any potential sex difference in fetal myostatin concentration.

In view of the above discussed knowledge gaps, we sought to assess whether cord blood myostatin concentrations are affected by GDM and fetal sex, the associations with fetal growth and fetal growth factors, and the role of testosterone in mediating potential sex difference in cord blood myostatin.

## Methods

### Study design, participants and specimens

We recruited pregnant women at the last prenatal visit before delivery at Mount Sinai Hospital in Toronto between January 2019 and February 2020. Participants met the following inclusion criteria: (1) maternal age 20-45 years; (2) single term pregnancy (gestational age ≥37 weeks); (3) Caucasian or Asian (to limit the potential confounding effects of ethnicity). Exclusion criteria were: (1) *in-vitro* fertilization; (2) any known birth defects or congenital anomalies; (3) family history of type 1 diabetes; (4) any major pre-pregnancy illnesses (e.g., type 1 or 2 or unknown diabetes, hypertension); (5) preeclampsia or other severe pregnancy complications. The study was approved by the Research Ethics Board of Mount Sinai Hospital (approval number: 18-0224-E). Written informed consent was obtained from all study participants.

All pregnant women underwent a 1-hour 50 g oral glucose challenge test between 24 and 28 weeks of gestation, and those who failed the test (1-h plasma glucose ≥7.8 mmol/L) were required to undertake a 75 g 2-h oral glucose tolerance test. GDM was diagnosed if any plasma glucose value was abnormal (fasting ≥5.3 mmol/L, 1-h ≥10.6 mmol/L, 2-h ≥9.0 mmol/L) according to the Canadian Diabetes Association’s diagnostic criteria (24). Mothers who had normal values in the 50 g oral glucose challenge test were considered to be euglycemic.

A total of 44 GDM and 66 euglycemic women were recruited. Data on sociodemographic characteristics and clinical risk factors were obtained by in-person interviews and reviews of medical charts using a standardized questionnaire by a trained research staff. Cord blood samples were collected by a trained research staff in the Research Centre for Women’s and Infants’ Health BioBank at Mount Sinai Hospital immediately following birth, kept on ice, and centrifuged within 2 hours after the specimen collection. Serum (without anti-coagulant) and EDTA plasma samples were stored in multiple aliquots at -80°C until assays.

### Biochemical assays

In our research lab (Dr Lye), cord plasma myostatin (pg/mL) was measured by an enzyme-linked immunosorbent assay (ELISA) kit (Cat# DGDF80, R & D Systems, Minneapolis, USA). Cord plasma IGF-2 (ng/mL) was measured by an ELISA kit (Cat# 22-IG2HU-E01, ALPCO Diagnostics, Salem, USA). Plasma proinsulin (pmol/L) was measured by an ELISA kit (Cat# 80-PINHUT-CH01, ALPCO Diagnostics, Salem, USA). In the clinical biochemistry laboratory of Mount Sinai Hospital, cord serum glucose (in mmol/L, 1 mmol/L=18 mg/dL) was determined by an enzymatic (hexokinase) method (Roche Diagnostics, Switzerland), serum insulin (in μU/mL, 1 μU/mL=6 pmol/L) and C-peptide (in ng/mL, 1 ng/mL= 333 pmol/L) were determined by chemiluminescence immunoassays (Roche Diagnostics, Switzerland), serum IGF-1 (ng/mL) was determined by a chemiluminescent immunoassay (Liaison XL, DiaSorin, Italy). Cord serum testosterone (nmol/L) was measured by a chemiluminescent immunoassay, and cross reactivity was 18% with 11-β-hydroxy-testosterone, less than 0.16% with estradiol and progesterone, and less than 6% for other testosterone-like hormones according to the manufacturer’s instructions (Roche Diagnostics, Switzerland). The limits of detection were 0.922 pg/mL for myostatin, 0.11 mmol/L for glucose, 0.2 mU/L for insulin, 0.01 ng/mL for C-peptide, 0.455 pg/mL for proinsulin, 10 ng/mL for IGF-1, 0.02 ng/mL for IGF-2, and 0.087 nmol/L for testosterone, respectively. Intra- and inter-assay coefficients of variation were in the ranges of 1.8-6.0% for myostatin, 0.5-1.3% for glucose, 0.8-4.9% for insulin, 0.5-2.3% for C-peptide, 4.0-9.7% for proinsulin, 2.37-8.5% for IGF-1, 3.1-7.2% for IGF-2, and 2.1-18.1% for testosterone, respectively. All biomarkers were assayed in duplicates, and the average values were taken. The assay technicians were blinded to the clinical characteristics of study subjects.

### Statistical analysis

The primary outcome was cord blood myostatin concentration. Birth weight z score was calculated according to the Canadian sex- and gestational age-specific birth weight standards (25). Mean±standard deviation (SD) or median (interquartile range) were presented for continuous variables. Frequency (percentage) was presented for categorical variables. Student’s t tests were conducted to compare continuous variables, and Chi-square tests or Fisher’s exact tests (where appropriate) were conducted to compare categorical variables between two groups. Pearson correlation coefficients were calculated to examine the correlations of cord blood myostatin with testosterone, fetal growth (birth weight z score) and fetal growth factors (insulin, C-peptide, proinsulin, IGF-1 and IGF-2). Log-transformed data were used for all cord blood biomarkers in t tests, correlation and regression analyses. Generalized linear regression was used to examine the determinants of cord blood myostatin. GDM status and fetal sex were the primary exposures of interest. Other covariates included maternal age, pre-pregnancy BMI (calculated from self-reported height and weight, kg/m^2^), ethnicity (Caucasian/Asian), education (university, yes/no), family history of type 2 diabetes (yes/no), smoking before pregnancy (yes/no), primiparity (yes/no), cesarean section (yes/no) and gestational age at delivery. Only a few mothers reported smoking (n=1) or alcohol drinking (n=2) during pregnancy, and thus not considered in data analyses. Covariates with P>0.2 that did not affect the comparisons were excluded in the parsimonious final regression models to obtain more stable effect estimates. In the presence of sex difference in cord blood myostatin concentrations, we examined the mediation effect of testosterone using the product (“Baron and Kenney”) method (26). P<0.025 was considered statistically significant in testing the primary hypothesis on the difference in cord blood myostatin concentrations by GDM status and fetal sex (Bonferroni correction for 2 tests). With Bonferroni correction for multiple tests, P<0.025 was considered statistically significant in examining the primary correlation of interest between cord blood myostatin and testosterone in sex-specific analyses. P<0.05 was considered statistically significant in other exploratory analyses. All data analyses were conducted in R Studio (Version 1.4.1717).

## Results

Compared with male *vs*. female newborns, the mothers were more likely to be Caucasian and had higher gestational weight gain (mean ± SD: 17.4 ± 7.2 *vs*. 13.2 ± 6.8 kg) (**Table 1**). As expected, males were heavier than females at birth (3458 ± 363 *vs*. 3239 ± 358 g). There were no significant differences in maternal age, pre-pregnancy BMI, GDM, primiparity, family history of type 2 diabetes, smoking before pregnancy, caesarean section and gestational age at delivery between male and female newborns.

**Table 1:** Characteristics of mothers and newborns by infant sex (n=110).

	Male (n=53)	Female (n=57)	P*
Mothers			
Age, years	34.0 ± 3.3	35.0 ± 3.9	0.15
>=35	20 (37.7)	29 (50.9)	0.23
Ethnicity			0.03
Caucasian	35 (66.0)	25 (43.9)	
Asian	18 (34.0)	32 (56.1)	
Primiparity	17 (32.1)	25 (44.6)	0.25
Education, less than university	9 (18.8)	7 (12.7)	0.57
Smoking before pregnancy	8 (15.4)	4 (7.1)	0.23
Pre-pregnancy BMI (kg/m^2^)	23.2 ± 4.16	24.3 ± 4.74	0.20
Overweight/obesity	13 (25.0)	22 (38.6)	0.19
Gestational weight gain (kg)	17.4 ± 7.2	13.2 ± 6.8	0.006
Family history of type 2 diabetes	12 (24.0)	13 (22.8)	1.00
Gestational diabetes mellitus	18 (34.0)	26 (45.6)	0.29
Newborns			
Cesarean section	37 (69.8)	40 (70.2)	1.00
Birth weight (g)	3458 ± 363	3239 ± 358	0.002
Birth weight z score	0.15 ± 0.82	-0.04 ± 0.81	0.23
Gestational age (weeks)	38.9 ± 0.79	38.9 ± 0.89	0.90

Data presented are mean ± SD or n (%).

*P values from Chi-square test or Student’s t test where appropriate.

Comparing GDM *vs*. euglycemic pregnancies, there were no significant differences in maternal age, education, primiparity, family history of type 2 diabetes, smoking before pregnancy, caesarean section, infant sex and gestational age (**Table 2**). Mothers with GDM had higher pre-pregnancy BMI (25.5 *vs*. 22.3 kg/m^2^) but lower gestational weight gain (12.9 *vs*. 16.8 kg), and were less likely to be Caucasian (39% *vs*. 65%). Unexpectedly, the newborns of GDM mothers had lower average birth weight than those of euglycemic mothers (3245 ± 369 *vs*. 3410 ± 365 g), partly due to more Asians (61% *vs*. 35%) who had lower birth weights than Caucasians (3250 ± 399 *vs*. 3422 ± 334 g). In Caucasian subjects (n=60), the newborns of GDM mothers had similar average birth weight *vs*. those of euglycemic mothers (3423 ± 332 *vs*. 3421 ± 339, P=0.99). In Asian subjects, the newborns of GDM mothers had lower average birth weight *vs*. those of euglycemic mothers (3133 ± 351 *vs*. 3387 ± 416, P=0.026).

**Table 2:** Characteristics of mothers and newborns by GDM status (n=110).

	GDM (n=44)	Non-GDM (n=66)	P*
Mothers			
Age, years	35.0 ± 4.1	34.3 ± 3.3	0.37
>=35	23 (52.3)	26 (39.4)	0.26
Ethnicity			0.01
Caucasian	17 (38.6)	43 (65.2)	
Asian	27 (61.4)	23 (34.8)	
Primiparity	18 (41.9)	24 (36.4)	0.71
Education, less than university	9 (21.4)	7 (11.5)	0.27
Smoking before pregnancy	4 (9.3)	8 (12.3)	0.76
Pre-pregnancy BMI (kg/m^2^)	25.5 ± 5.2	22.3 ± 3.3	<0.001
Overweight/obesity	21 (47.7)	12 (18.5)	0.002
Gestational weight gain (kg)	12.9 ± 7.1	16.8 ± 7.0	0.02
Family history of type 2 diabetes	14 (32.6)	11 (17.2)	0.11
Newborns			
Cesarean section	27 (61.4)	50 (75.8)	0.16
Sex, male	18 (40.9)	35 (53.0)	0.29
Birth weight (g)	3245 ± 369	3410 ± 365	0.02
Birth weight z score	-0.12 ± 0.85	0.16 ± 0.77	0.16
Gestational age (weeks)	38.8 ± 0.90	39.0 ± 0.80	0.45

Data presented are mean ± SD or n (%).

*P values from Chi-square test or Student’s t test where appropriate.

GDM=Gestational diabetes mellitus.

**Figure 1:**
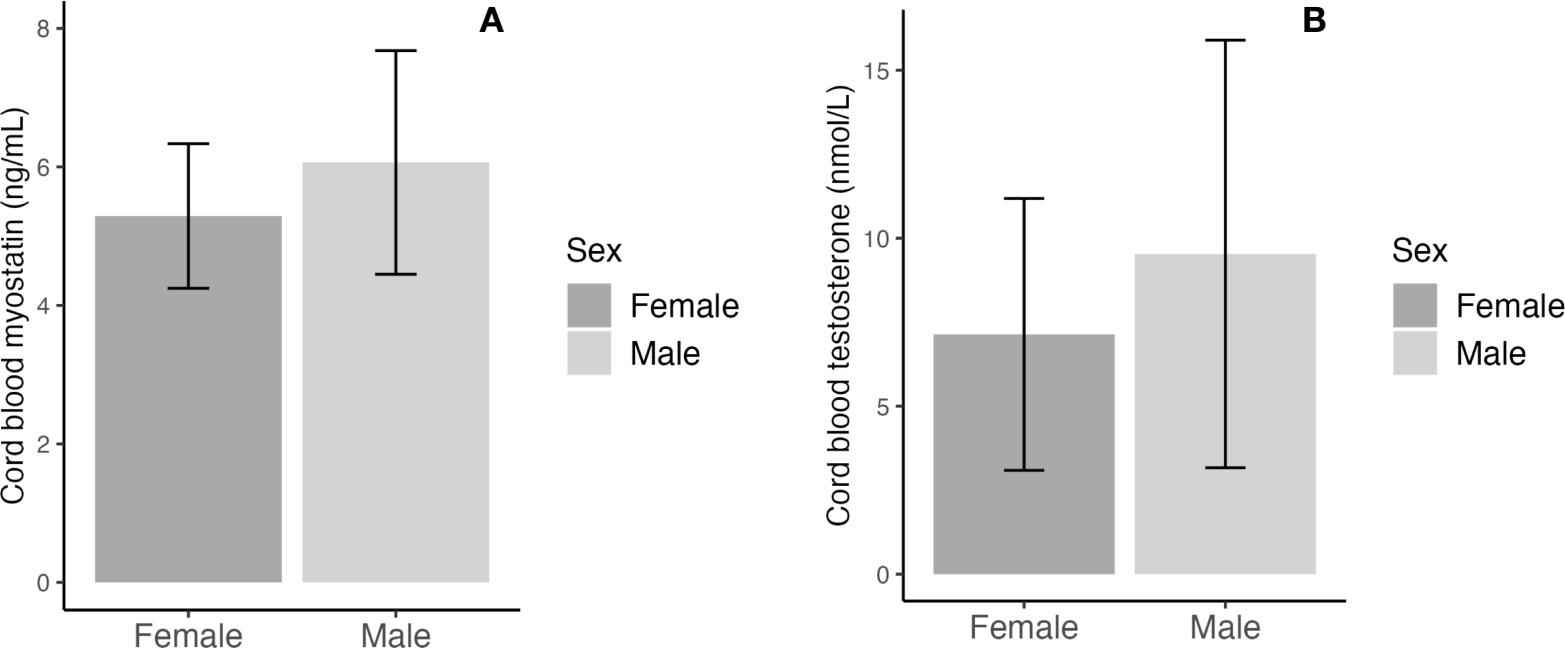
Sex differences in cord blood concentrations of myostatin **(A)** and testosterone **(B)**; the error bars represent the mean and 95% confidence intervals.

Adjusting for maternal and infant characteristics, male newborns had significantly higher cord blood concentrations of myostatin (mean: 6.07 *vs*. 5.29 ng/mL, adjusted P=0.006) and testosterone (9.53 *vs*. 7.14 nmol/L, adjusted P=0.017) (**Table 3** and **Figure 1**). As expected, female newborns tended to have higher cord blood concentrations of insulin (102.8 *vs*. 62.9 pmol/L, adjusted P=0.074) and proinsulin (23.3 *vs*. 17.7 pmol/L, adjusted P=0.066). Cord blood glucose/insulin ratio - a surrogate indicator of fetal insulin sensitivity (14, 27), was higher in males *vs*. females (7.87 *vs*. 5.46, adjusted P=0.048). Cord blood myostatin and testosterone concentrations were similar in Caucasian *vs*. Asian newborns (all P>0.5, data not shown). There were no significant interactions between fetal sex and GDM status, or between fetal sex and ethnicity in relation to cord blood myostatin or testosterone (all P>0.1).

**Table 3:** Cord blood concentrations of myostatin, testosterone and fetal growth factors comparing male *vs*. female newborns.

	Males (n=53)	Females (n=57)	Crude P*	Adjusted P*
Myostatin (ng/mL)	6.07 ± 1.62	5.29 ± 1.04	0.009	0.006
	5.77 (4.86, 7.15)	5.26 (4.42, 5.99)		
Testosterone (nmol/L)	9.53 ± 6.36	7.14 ± 4.05	0.005	0.017
	7.70 (6.05, 9.00)	6.20 (4.70, 7.80)		
Glucose (mmol/L)	3.35 ± 0.67	3.44 ± 0.75	0.54	0.66
	3.20 (2.90, 3.70)	3.40 (2.90, 3.95)		
Insulin (pmol/L)	62.9 ± 36.4	102.8 ± 102.4	0.014	0.074
	58.0 (42.0, 71.0)	80.0 (47.0, 105.0)		
Glucose/Insulin ratio	7.87 ± 5.46	5.46 ± 3.69	0.008	0.048
(mg/dL/μU/mL)^£^	6.16 (5.12, 8.77)	4.75 (3.60, 6.93)		
Proinsulin (pmol/L)	17.7 ± 8.74	23.3 ± 14.6	0.009	0.066
	15.0 (12.6, 17.5)	18.9 (14.6, 26.1)		
C-peptide (pmol/L)	410 ± 145	453 ± 228	0.41	0.30
	369 (324, 465)	407 (317, 518)		
IGF-1 (ng/mL)	110 ± 29	111 ± 29	0.93	0.29
	105 (90, 136)	109 (91, 134)		
IGF-2 (ng/mL)	387 ± 59	393 ± 71	0.74	0.96
	366 (343, 410)	376 (343, 448)		

Data presented are Mean ± SD and Median (Q25, Q75).

IGF-1, insulin-like growth factor-1; IGF-2, insulin-like growth factor-2.

Glucose, insulin, C-peptide, IGF-1 and testosterone were measured in cord blood serum, myostatin, proinsulin and IGF-2 were measured in cord blood EDTA plasma samples.

^£^ Glucose/Insulin ratio in mg/dL/mU/mL (glucose: 1 mmol/L=18 mg/dL; insulin: 1 μU/mL=6 pmol/L); higher cord blood glucose/insulin ratios indicate higher fetal insulin sensitivity.

*Crude P values were from t-tests in log-transformed biomarker data. Adjusted P values were from generalized linear models in the comparisons of log-transformed biomarker data adjusting for maternal (age, ethnicity, pre-pregnancy BMI, gestational weight gain, gestational diabetes mellitus) and neonatal (cesarean section) characteristics; other factors were excluded since they were similar and did not affect the comparisons (all P>0.2).

Tests for interaction between fetal sex and GDM in relation to cord blood biomarkers, all P>0.2 (data not shown).

Cord blood myostatin concentrations were similar in GDM *vs*. euglycemic pregnancies (5.50 *vs*. 5.77 ng/mL, adjusted P=0.28, **Table 4**). There were no significant differences in cord blood concentrations of testosterone, IGF-1 and IGF-2 comparing the newborns of GDM *vs*. euglycemic mothers. Similarly, in male newborns, there were no significant differences in cord blood concentrations of myostatin (5.94 *vs*. 6.13 ng/mL, adjusted P=0.57) and testosterone (9.74 *vs*. 9.43 nmol/L, adjusted P=0.56) comparing GDM *vs*. euglycemic pregnancies. The newborns of GDM mothers tended to have higher insulin (120.4 *vs*. 60.1 pmol/L, adjusted P=0.07) and proinsulin (24.5 *vs*. 18.0 pmol/L, adjusted P=0.14) concentrations, although the differences did not reach statistical significance. Similarly, cord blood concentrations tended to be higher for insulin (90.5 *vs*. 52.2 pmol/L, P=0.026) and C-peptide (462 *vs*. 385 pmol/L, P=0.08) comparing GDM and euglycemic pregnancies in male newborns,

**Table 4:** Cord blood concentrations of myostatin, testosterone and fetal growth factors comparing GDM *vs*. euglycemic pregnancies.

	GDM (n=44)	Control (n=66)	Crude P*	Adjusted*
Myostatin (ng/mL)	5.50 ± 1.37	5.77 ± 1.42	0.29	0.28
	5.36 (4.44, 6.30)	5.57 (4.56, 6.57)		
Testosterone (nmol/L)	8.77 ± 6.74	7.98 ± 4.38	0.70	0.89
	6.80 (5.30, 8.10)	7.40 (5.20, 8.80)		
Glucose (mmol/L)	3.64 ± 0.83	3.24 ± 0.58	**0.01**	0.13
	3.60 (3.10, 4.10)	3.20 (2.80, 3.60)		
Insulin (pmol/L)	120.4 ± 114.3	60.1 ± 29.7	**0.002**	0.07
	80.0 (64.0, 144.0)	58.0 (41.5, 74.3)		
Glucose/Insulin ratio	5.53 ± 4.22	7.39 ± 5.05	**0.02**	0.25
(mg/dL/mU/mL) ^£^	4.34 (2.73, 7.13)	5.80 (4.92, 7.94)		
Proinsulin (pmol/L)	24.5 ± 16.3	18.0 ± 8.2	0.04	0.14
	18.7 (13.9, 26.4)	15.6 (12.7, 21.0)		
C-peptide (pmol/L)	494 ± 261	393 ± 120	0.054	0.33
	460 (324, 620)	375 (313, 462)		
IGF-1 (ng/mL)	107.1 ± 29.3	112.6 ± 29.9	0.35	0.71
	104 (89, 122)	111 (91, 137)		
IGF-2 (ng/mL)	392 ± 67	388 ± 65	0.80	0.57
	366 (343, 448)	375 (342, 425)		

Data presented are Mean ± SD and Median (Q25, Q75).

IGF-1, insulin-like growth factor-1; IGF-2, insulin-like growth factor-2.

Glucose, insulin, C-peptide, IGF-1 and testosterone were measured in cord blood serum, myostatin, proinsulin and IGF-2 were measured in cord blood EDTA plasma samples.

^£^Glucose/Insulin ratio in mg/dL/mU/mL (glucose: 1 mmol/L=18 mg/dL; insulin: 1 mU/mL=6 pmol/L); higher cord blood glucose/insulin ratios indicate higher fetal insulin sensitivity.

*Crude P values were from t-tests in log-transformed biomarker data. Adjusted P values were from generalized linear models in the comparisons of log-transformed biomarker data between the two groups adjusting for maternal (age, ethnicity, pre-pregnancy BMI, gestational weight gain and family history of diabetes) and neonatal (fetal sex, cesarean section) characteristics; other factors were excluded since they were similar and did not affect the comparisons (all P>0.2). P values in bold: P < 0.025.

**Figure 2:**
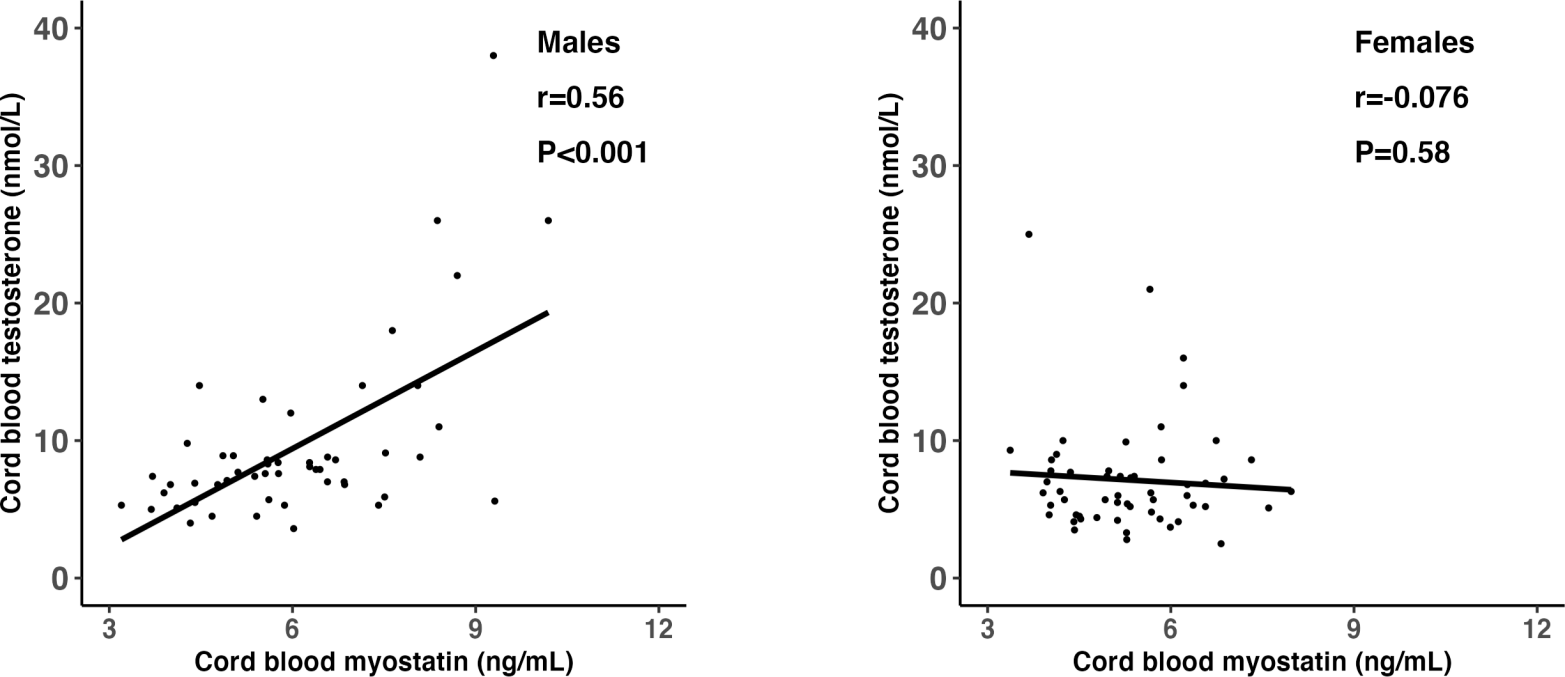
Scatter plots illustrating the differential correlations (r) between cord blood myostatin and testosterone in males and females; Fisher’s Z test for difference in r (correlation coefficient): P<0.001.

Adjusting for gestational age at blood sampling, cord blood myostatin was positively correlated with testosterone in males (r=0.56, P <0.001), but not in females (r=-0.076, P=0.58) (Fisher’s z test for difference in correlation coefficients, P<0.001) (**Table 5** and **Figure 2**). In the total study sample, cord blood myostatin was negatively correlated with IGF-2 (r=-0.23, P=0.02), but not correlated with birth weight (z score), IGF-1, insulin, proinsulin or C-peptide. Cord blood myostatin was not correlated with glucose/insulin ratio in males (P=0.46) or females (P=0.35).

**Table 5:** Correlations of cord blood myostatin with testosterone, birth weight (z), glucose/insulin ratio and fetal growth factors.

	All		Males		Females		*P for difference
	r	P	r	P	r	P	
Myostatin with:							
Testosterone	0.34	<0.001	0.56	<0.001	-0.076	0.58	<0.001
Birth weight (z)	0.06	0.53	-0.024	0.87	0.10	0.44	0.51
Insulin	-0.14	0.25	-0.16	0.35	0.04	0.83	0.41
Glucose/Insulin	0.071	0.57	0.13	0.46	-0.17	0.35	0.28
Proinsulin	-0.01	0.90	0.017	0.90	0.09	0.51	0.71
C-peptide	-0.11	0.27	-0.15	0.32	-0.05	0.71	0.64
IGF-1	-0.05	0.60	-0.21	0.14	0.15	0.28	0.07
IGF-2	-0.23	0.02	-0.18	0.20	-0.29	0.03	0.55
Testosterone with:							
Birth weight (z)	-0.075	0.45	-0.14	0.33	0.21	0.13	0.84
Insulin	-0.26	0.03	-0.43	0.011	0.11	0.55	0.053
Glucose/Insulin	0.18	0.15	0.33	0.054	-0.19	0.30	0.06
Proinsulin	-0.10	0.32	-0.15	0.31	0.044	0.75	0.32
C-peptide	-0.07	0.47	-0.32	0.025	0.16	0.26	0.025
IGF-1	-0.10	0.30	-0.24	0.10	0.03	0.84	0.21
IGF-2	0.28	0.004	0.22	0.13	0.37	0.005	0.35

Data presented are Pearson partial correlation coefficients adjusting for gestational age at delivery/cord blood sampling. Log-transformed data were used for all biomarkers in the partial correlation analyses.

IGF-1, insulin-like growth factor-1; IGF-2, insulin-like growth factor-2.

*P values in Fisher’s z tests for differences in correlation coefficients in males and females.

Adjusting for gestational age at blood sampling, cord blood testosterone was positively correlated with IGF-2 (r=0.28, P=0.004), but not correlated with IGF-1 (r=-0.10, P=0.30) or birth weight (r=-0.075, P=0.45) in the total study sample (**Table 5**). Cord blood testosterone was negatively correlated with insulin in males (r=-0.43, P=0.011), but not in females (r=0.11, P=0.55) (Fisher’s z test for difference in correlation coefficients, P=0.053). Similarly, testosterone was negatively correlated with C-peptide (r=-0.32, P=0.025) in males, but not in females (r=0.16, P=0.26) (Fisher’s z test for difference in correlation coefficients, P=0.025).

There were no significant differences in the correlations of cord blood myostatin and testosterone with birth weight and fetal growth factors by GDM status or ethnicity (data not shown).

In the total study sample, birth weight z score was positively correlated with cord blood proinsulin (r=0.24, P=0.01) and IGF-1 (r=0.31, P=0.001), and tended to be positively correlated with C-peptide (r=0.17, P=0.08), but not correlated with IGF-2 (r=0.01, P=0.94).

Overall, male sex was associated with a 0.60 increase in cord blood myostatin concentration z score (P=0.002). Mediation analysis demonstrated that cord blood testosterone could explain a 0.18 increase in cord blood myostatin z score (30.0%, P=0.039) in males *vs*. females (**Table 6**). Cord blood testosterone or myostatin could not explain the sex difference in cord blood glucose/insulin ratio or birth weight (all P>0.1 for mediation effect, results not shown).

**Table 6:** Mediation analysis in the association of fetal sex with cord blood myostatin (n=110).

	Myostatin (z score)	
	β (95% CI)	P
Fetal sex, male	0.60 (0.23, 0.96)	**0.002**
*Mediation by testosterone	0.18 (0.072, 0.28)	**0.039**
Maternal age (per SD)	0.15 (-0.03, 0.33)	0.10

Data (β) presented are the standardized changes in cord blood myostatin (z score) from generalized linear models. Only fetal sex and maternal age were predictive of cord blood myostatin at P<0.2; other maternal and infant characteristics did not affect the comparisons and were thus not included in the final model. The SDs for calculating the z scores were 1.40 ng/mL for myostatin, 5.40 nmol/L for testosterone and 3.63 years for maternal age.

*The mediation effect presented is the change (95% CI) in cord blood myostatin (z score) per SD increment in cord blood testosterone that could account for the effect of fetal sex on cord blood myostatin. P values in bold: P < 0.05.

## Discussion

### Main findings

Our study is the first to demonstrate that GDM does not affect cord blood myostatin concentration, whereas fetal sex does. Cord blood myostatin concentrations were significantly higher in males *vs*. females. Approximately 30% of the sex difference in cord blood myostatin concentrations can be explained by testosterone. Interestingly, cord blood testosterone and myostatin was positively correlated in males only, suggesting a male-specific androgen up-regulation of myostatin secretion in early life in humans.

### GDM and cord blood myostatin

Cord blood myostatin concentrations were similar in GDM *vs*. controls. Skeletal muscle is the dominant source of circulating myostatin (1); skeletal muscle-specific expression of myostatin is about 50-100 fold higher than adipose tissue-specific expression (28). Whether GDM affects skeletal muscle mass remains controversial; similar or lower lean mass has been reported in the neonates of GDM *vs*. controls (29, 30). Although GDM has been associated with increased fat mass in the offspring (29, 30), myostatin expression in both visceral and subcutaneous fat was similar in obese *vs*. lean subjects (11), and circulating myostatin concentration does not appear to be correlated with fat mass (12).

### Cord blood myostatin, fetal sex and testosterone

We observed significantly higher cord blood myostatin concentrations in males *vs*. females. In contrast, a smaller study (n=83) reported no significant difference in cord blood myostatin concentrations in males *vs*. females (18). The reasons for the discrepant findings may be partly due to the differences in sample size and detection method (Sandwich ELISA in our study *vs*. competitive ELISA kit in their study). Our study is consistent with an adult study reporting higher myostatin concentrations in males *vs*. females (4.3 ng/mL *vs*. 3.3 ng/mL) using the same ELISA kit as in our study (R&D Systems) (12).

Interestingly, cord blood myostatin was positively correlated with testosterone in males but not in females. Testosterone is an anabolic hormone promoting protein synthesis and skeletal muscle growth (19). In contrast, myostatin inhibits skeletal muscle growth (1, 2). Our data support the hypothesis that testosterone may up-regulate myostatin (and thus may counteract the effect of testosterone) in males. This observation is in line with a study in male mice reporting that the inhibition of testosterone production or androgen receptor signaling could down-regulate myostatin gene expression and protein synthesis in androgen responsive muscles (31). An adult study showed that both testosterone and myostatin concentrations were higher in young *vs*. old men, and testosterone treatment resulted in higher myostatin concentrations (23), indicating that testosterone may up-regulate myostatin secretion in men. Our data suggest that testosterone may up-regulate fetal myostatin secretion in males, and may partly mediate the higher fetal (cord blood) myostatin concentrations in males in humans.

### Myostatin, testosterone and fetal insulin sensitivity

Our study confirmed that GDM and female sex were associated with lower fetal insulin sensitivity as indicated by lower cord blood glucose/insulin ratios and higher insulin and proinsulin concentrations (14, 15). Neither myostatin nor testosterone could explain the differences in glucose/insulin ratios by fetal sex or GDM in mediation analyses, suggesting neither may explain such differences. GDM was not associated with cord blood testosterone, consistent with a previous study (32). Testosterone tended to be positively correlated with fetal insulin sensitivity (glucose/insulin) in males (P=0.054). This is in line with a previous study reporting that testosterone replacement therapy improved insulin resistance in adult men (22).

### Myostatin and testosterone in relation to fetal growth and fetal growth factors

We observed a negative correlation between cord blood myostatin and IGF-2, but no correlation with IGF-1 or birth weight. Myostatin appears to be regulated by growth hormone in hypophysectomised mice (33) and hypopituitary adults (34), suggesting myostatin may play a role in fetal growth. A previous study reported an inverse correlation between cord blood myostatin and birth weight in 83 newborns (r=-0.40, P=0.001) (18). The reasons for the different findings may be partly due to the differences in study population and detection methods (Sandwich *vs*. competitive ELISA). Their study included 23 large-for-gestational-age (LGA, birth weight z score >2) and 60 appropriate-for-gestational-age infants (birth weight z score from -1 to 1) (18), and the larger differences in birth weight may render a greater power to identify a significant correlation between cord blood myostatin and birth weight. None of our newborns could be identified as LGA if we used the same definition as in their study. On the other hand, we did observe a negative correlation between cord blood myostatin and IGF-2, suggesting a possible negative effect on fetal growth. IGF-2 plays a pivotal role in fetal growth (35, 36). Our observation is in line with two animal studies reporting that IGF-2 expression was greater in mice with myostatin mutation (37), and IGF-2 expression was inhibited in myoblast cultures with treatment of recombinant myostatin (38). Overall, our data are somewhat uncertain concerning the role of myostatin in fetal growth. We could not rule out the possibility of a false negative finding, and larger studies are warranted to clarify the role of myostatin in fetal growth.

Testosterone was positively correlated with IGF-2, but not correlated to IGF-1 or birth weight. IGF-2 is a fetal growth factor important for early embryonic fetal growth, and its correlation with birth weight tends to be much weaker than IGF-1 (35, 36). We failed to detect a positive correlation between IGF-2 and birth weight, probably due to the relative small sample size. The lack of correlation between cord blood testosterone and birth weight is consistent with the results in a previous study (32). As expected, birth weight was positively correlated with cord blood proinsulin, C-peptide and IGF-1, consistent with the findings in previous studies (39, 40).

Interestingly, we observed a negative correlation between cord blood testosterone and insulin or C-peptide in males but not in females. More studies from other independent cohorts are warranted to confirm this novel observation suggesting that testosterone may play a sex dimorphic role in insulin secretion during fetal life in humans.

### Limitations

There are some study limitations. Firstly, the modest sample size allowed for the detection of modest/large differences, and was underpowered to detect small differences. With the study sample size (44 GDM, 66 controls; 53 males, 57 females) with alpha error at 0.025, we had a power of >=91% to detect a 0.7 SD or greater difference in cord blood myostatin concentrations between GDM and controls, or between males and females. The study power was >78% to detect an absolute correlation coefficient of 0.4 or greater in sex-specific analyses with alpha error at 0.025. Secondly, cord serum testosterone was measured by chemiluminescence immunoassay rather than mass spectrometry - the golden standard (much more costly) method. Due to cross reactivity with testosterone-like molecules, the observed cord serum testosterone concentrations could have been inflated to some extent. However, such noise random variations would only tend to decrease the probability of detecting a significant association. Lastly, the observational nature of the study precluded the possibility of conclusive causal inference.

In conclusion, GDM does not affect cord blood myostatin concentration, but fetal sex does. The higher myostatin concentrations in males may be partly mediated by testosterone. The male-only positive correlation between cord blood testosterone and myostatin suggests a male-specific role of androgen in up-regulating fetal myostatin secretion in humans.

## Data availability statement

The datasets presented in this article are not readily available because Access to deidentified research data must be approved by the research ethics board. Requests to access the datasets should be directed to zcluo@lunenfeld.ca.

## Ethics statement

The studies involving human participants were reviewed and approved by Research Ethics Board of Mount Sinai Hospital University of Toronto. The patients/participants provided their written informed consent to participate in this study.

## Author contributions

RH, LB, ZP, KM, JK, SL and Z-CL conceived the study. RH, MK, KM, JK, SL and Z-CL contributed to the acquisition of research data. RH conducted the literature review, data analysis and drafted the article. All authors contributed in revising the article critically for important intellectual content, and approved the final version for publication. Z-CL is the guarantor of this work, has full access to all the data in the study and takes responsibility for the integrity of the data and the accuracy of the data analysis. All authors approved the submitted version.
